# Quorum Sensing-Mediated Lipid Oxidation Further Regulating the Environmental Adaptability of *Aspergillus ochraceus*

**DOI:** 10.3390/metabo13040491

**Published:** 2023-03-29

**Authors:** Jing Gao, Huiqing Liu, Zhenzhen Zhang, Zhihong Liang

**Affiliations:** 1 College of Food Science and Nutritional Engineering, China Agricultural University, Beijing 100083, China; 2 Key Laboratory of Safety Assessment of Genetically Modified Organism (Food Safety), Ministry of Agriculture, Beijing 100083, China; 3 Beijing Laboratory for Food Quality and Safety, China Agricultural University, Beijing 100083, China

**Keywords:** quorum sensing, cell communication, oxylipin, G-protein, lipid metabolism, multiomics

## Abstract

Quorum sensing (QS) is a cellular strategy of communication between intra- and inter-specific microorganisms, characterized by the release of quorum sensing molecules (QSMs) that achieve coordination to adaptation to the environment. In *Aspergillus*, lipids carry population density-mediated stresses, and their oxidative metabolite oxylipins act as signaling to transmit information inside cells to regulate fungal development in a synchronized way. In this study, we investigated the regulation of density-dependent lipid metabolism in the toxigenic fungi *Aspergillus ochraceus* by the oxidative lipid metabolomics in conjunction with transcriptomics. In addition to proven hydroxyoctadecadienoic acids (HODEs), prostaglandins (PGs) also appear to have the properties of QSM. As a class of signaling molecule, oxylipins regulate the fungal morphology, secondary metabolism, and host infection through the G protein signaling pathway. The results of combined omics lay a foundation for further verification of oxylipin function, which is expected to elucidate the complex adaptability mechanism in *Aspergillus* and realize fungal utilization and damage control.

## 1. Introduction

*Aspergillus* is a genus of filamentous saprophytic fungi which contains four subgenera, 27 sections, and 339 species, and ubiquitously distributed in natural habitats [1]. Some *Aspergillus* species, such as *A. nidulans*, *A. niger*, *A. oryzae*, *A. terreus* and *A. sojae,* are generally recognized as safe (GRAS) by the Food and Drug Administration (FDA), as eukaryotic cell factories used in food, medicine and other fields of industrialization fermentation and biotechnology. However, some *Aspergillus* species are terrible food spoilage fungi and/or opportunistic human pathogens which can produce harmful secondary metabolite mycotoxins and pose a serious threat to animal and human health [2]. *A. ochraceus* is the representative species and a model mycotoxin-producing strain in *Aspergillus* section *Circumdati*, which widely contaminates raw materials and processed foods, spatiotemporally involving plant, harvest, process, storage and sale, causing foods' sensory quality reduction, physical damage and chemical composition destruction [3,4]. On the other hand, some evidence suggests that *A. ochraceus* can cause onychomycosis [5], allergic bronchopulmonary aspergillosis [6], COVID-19 associated pulmonary aspergillosis [7], and otomycosis [8], and its mycelial proteins have allergenic potency [9]. In 1965, Van et al. isolated *A. ochraceus* from sorghum grain and found that the toxicity of moldy grains was caused by a new toxic metabolite—Ochratoxin A (OTA) [10], which has hepatotoxicity, nephrotoxicity, enterotoxicity, immunotoxicity, neurotoxicity, etc. [11], and it was classified as a Group 2B carcinogen (a possible human carcinogen) by the International Agency for Research on Cancer (IARC) in 1993 [12].

The reproduction of toxigenic fungi and the biosynthesis of mycotoxins at different stages are significantly influenced by external environmental factors, signaling molecules, and trans-kingdom communication. With the increase of population density in a limited living space, resource shortages will cause inter- and intra-specific competition for nutrients, oxygen etc.; on the other hand, single cells will communicate and cooperate in a more “holistic” form, such as a biofilm, in order to protect individuals against environmental pressures. This perception of a population signal was first defined as quorum sensing (QS) in 1994 [13], and is one of the main mechanisms for microbial communication through secreted and sensed hormone-like small molecular compounds known as quorum-sensing molecules (QSMs) which play a role in monitoring population density and regulating the physiology of fungi to adapt to environmental changes [14]. Farnesol found the earliest proven fungal QSM in *Candida albicans* [15], and currently verified QSM include alcohols, oxylipins, small molecule peptide pheromones, and certain volatile substances [16].

**Figure 1:**
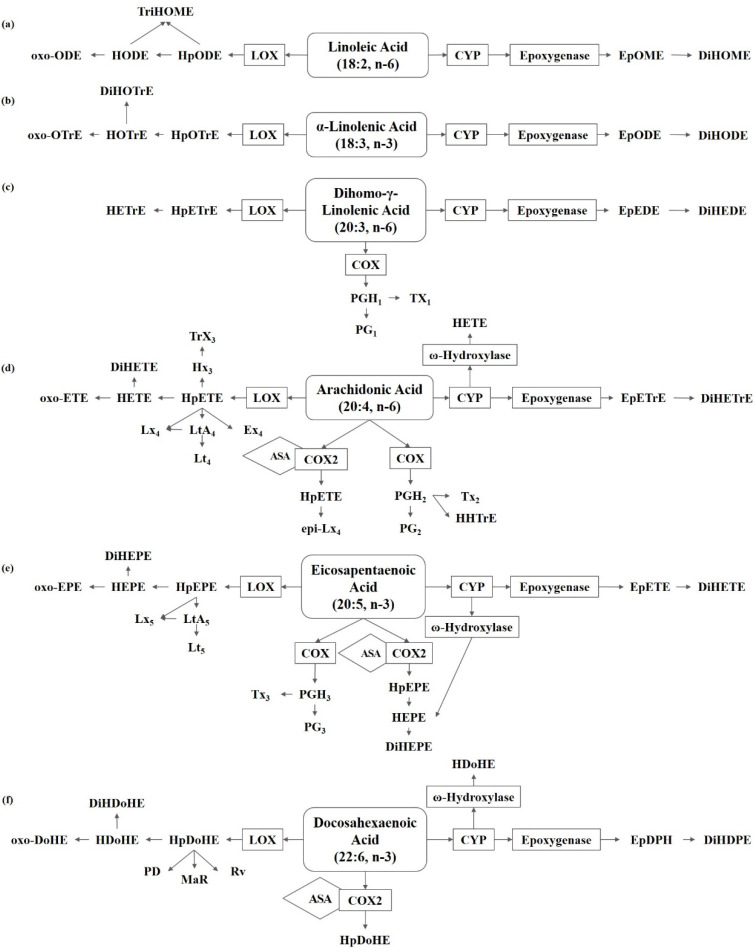
Metabolism of polyunsaturated fatty acids in fungi. (**a**) Linoleic acid (LA) derived oxylipins. (**b**) α-Linolenic acid (ALA) derived oxylipins. (**c**) Dihomo-γ-linolenic acid (DGLA) derived oxylipins. (**d**) Arachidonic acid (ARA) derived oxylipins. (**e**) Eicosapentaenoic acid (EPA) derived oxylipins. (**f**) Docosahexaenoic acid (DHA) derived oxylipins. ASA, acetylsalicylic acid; COX, cyclooxygenase; CYP, cytochrome P450; DiHDoHE, dihydroxy-docosahexaenoic acid; DiHDPE, dihydroxy-docosapentaenoic acid; DiHEDE, dihydroxy-eicosadienoic acid; DiHEPE dihydroxy-eicosapentaenoic acid; DiHETE, dihydroxy-eicosatetraenoic acid; DiHETrE, dihydroxy-eicosatrienoic acid; DiHODE, dihydroxy-octadecadienoic acid; DiHOME, dihydroxy-octadecenoic acid; DiHOTrE, dihydroxy-octadecatrienoic acid; EpDPE, epoxy-docosapentaenoic acid; EpEDE, epoxy-eicosadienoic acid; EpETE, epoxy-eicosatetraenoic acid; EpETrE, epoxy-eicosatrienoic acid; EpODE, epoxy-octadecadienoic acid; EpOME, epoxy-octadecenoic acid; Ex, eoxin; HDoHE, hydroxyl-docosahexaenoic acid; HEPE, hydroxyl-eicosapentaenoic acid; HETE, hydroxyl-eicosatetraenoic acid; HETrE, hydroxy-eicosatrienoic acid; HHTrE, hydroxyl-heptadecatrienoic acid; HODE, hydroxy-octadecadienoic acid; HOTrE, hydroxy-octadecatrienoic acid; HpDoHE, hydroperoxy-docosahexaenoic acid; HpEPE, hydroperoxy-eicosapentaenoic acid; HpETE, hydroperoxy-eicosatetraenoic acid; HpETrE, hydroperoxy-eicosatrienoic acid; HpODE, hydroperoxy-octadecadienoic acid; HpOTrE, hydroperoxy-octadecatrienoic acid; Hx, hepoxilin; LOX, lipoxygenase; Lt, Leukotriene; Lx, lipoxin; MaR, maresin; oxo-DoHE, oxo-docosahexaenoic acid; oxo-EPE, oxo-eicosapentaenoic acid; oxo-ETE, oxo-eicosatetraenoic acid; oxo-ODE, oxo-octadecadienoic acid; oxo-OTrE, oxo-octadecatrienoic acid; PD, protectin; PGEM, prostaglandin E metabolite; Rv, resolvin; TriHOME, trihydroxy-octadecenoic acid; Trx, trioxilin; Tx, thromboxane.

Oxylipin is a broad family of enzymatic or non-enzymatic oxidation secondary metabolites of monounsaturated fatty acids (MUFAs) and/or polyunsaturated fatty acids (PUFAs). Free fatty acids (FFAs) are cleaved from FAs and diversified by length and degree of unsaturation, which may be incorporated into complex membrane lipids (phospholipids, glycolipids and sterol), and provide the substrate to oxylipin synthesis: oleic acid (OA), linoleic acid (LA), α-linolenic acid (ALA), dihomo-γ-linolenic acid (DGLA), arachidonic acid (ARA), eicosapentaenoic acid (EPA), and docosahexaenoic acid (DHA) [17]. The study of fungal oxylipins followed the discovery of lipid hydroperoxides in *A. parasiticus* [18], and the precocious sexual inducer (psi) factor is one of the earliest extracellular signaling molecules of lipid oxidation [19]. The enzymatic production of oxylipin in vivo occurs mainly through oxidase pathways. The lipoxygenase (LOX) family is conserved in higher plants, animals and fungi, requires manganese as the cofactor, and catalyzes the formation of hydroperoxy FAs, and then rapidly converts to hydroxy FAs. The monooxygenase family is much more prevalent in fungi than LOX and cyclooxygenase (COX) homologs, which is a broader class of specialized cytochrome P450 enzymes (CYP) that have epoxygenase or ω-hydroxylase activity. The dioxygenase (DOX) family contains a catalytic domain that is often fused to a functional cytochrome P450 at the C-terminal end in the shape of DOX-CYP fusion enzymes, including linoleate dioxygenase (LDS) and fungal unique dioxygenases-cytochrome P450 (DO-P450), which are also known as psi producing oxygenases (PPO). Different mammals, plants, and microorganisms have evolutionarily related oxidases and similar process of oxylipin synthesis (Figure 1), which have been professionally summarized by Gabbs et al. (2015), Oliw EH (2021), and An JU (2021) [20,21,22]. At the same time, oxylipin is also a class of recipient of oxidative stress, such as reactive oxygen species (ROS), and the non-enzymatic production of oxylipins are usually used as biomarkers in medicine [17].

Interspecific coexistent oxylipins have similar structures and functions, which can transmit extracellular information between cells by binding to membrane receptors such as G-protein coupled receptors (GPCRs), and through intracellular effectors such as second messenger to modify transcription factors. The expression of related genes will then be regulated and ultimately affect fungal behaviors [23,24]. *Aspergillus* has great changes in morphological switch, reproduction, secondary metabolism, etc. under different population densities, which was related to the diversified types and quantities of QSMs under different degrees of environmental pressures, and this was also confirmed by exogenous addition experiments. For example, hydroxyoctadecadienoic acids (HODEs) are produced by the hydroxylation of carbon atoms at different sites of linoleic acid catalyzed by different LOXs; 9-HODE and 13-HODE alter reproductive formation and secondary metabolism in *A. parasiticus*, *A. nidulans* and *A. flavus* [25,26,27]. In *A. ochraceus* at low density, there are fewer conidia but more OTA and the 9-HODE/13-HODE ratio is higher than high density. The addition of 9-HODE inhibited conidiospores formation and promoted OTA synthesis, while 13-HODE had the complete opposite effect [28]. HODEs were proved to be a class of ligand of GPCR in *Aspergillus*, which via the intracellular cAMP effector influence the conversion between sexual and asexual, vegetative growth, secondary metabolism, pathogenicity, infectivity, and resistance [27]. It is worth noting that prostaglandins (PGs) acting as the ligand of G2A (a class of GPCR related to immunity system in mammals) also exist in minuscule amounts in *Aspergillus*, but its precise mechanism remains to be further elucidated [29].

Due to the necessity of fungal pollution prevention and control, understanding the influence factors and regulation of *A. ochraceus* growth and OTA biosynthesis is crucial, and there have been studies on the QS mechanism and G-protein signaling pathway in *Aspergillus*. However, no density-dependent analysis of the oxidized lipidomics of *A. ochraceus* has been conducted, and no definitive QSM has been identified.

In our study, we first qualitatively and quantitatively identified the oxidized lipid metabolites in typical high and low densities of *A. ochraceus* and screened the different metabolites affected by population density. Transcriptomics was used to enrich differentially expressed genes (DEGs) into specific pathways and combined with functional annotations of differential lipid metabolites to explore the causes of fungal behavior changes related to oxylipins, in addition to the mechanism of density-mediated stress that affects fungal behaviors. This work is expected to provide the *A. ochraceus* model for studies on the mechanism of *Aspergillus* quorum sensing, which reflects the comprehensive environmental stress caused by density. In addition, it will analyse the metabolism of oxylipins, which is expected to contribute to the prevention and control of fungal contamination by interrupting the cell communication.

## 2. Materials and Methods

### 2.1. Fungal Strain and Culture Conditions

The *Aspergillus ochraceus* strain (CGMCC No. 3.4412; GenBank GCA_004849945.1; BioProject ID PRJNA264608) was purchased from the Institute of Microbiology of the Chinese Academy of Sciences and preserved in glycerol at −80 °C in the lab. *Aspergillus* spp. strains were activated on a potato dextrose agar (PDA) medium (Zolarbio, Beijing, China) at 28 °C for 7 days in the dark and the process was repeated three times.

In general, *A. ochraceus* that grows in PDA medium for 5 days will produce enough spores to cover the plates, which is suitable for scraping and preparing spore suspensions. We used a sterilized hole punch to gently take a 1 cm diameter PDA medium with spores, and placed it in a centrifuge tube containing 1 mL of sterile saline that contained 0.05% Tween 80, then shaking it vigorously for 30 s to resuspend the spores. The spore concentration was counted using a haemacytometer, and each count was repeated three times in parallel, with the final concentration of the spore suspension being adjusted as needed. Based on the previous series of density gradient (10^1^–10^7^ spores/mL) experiments and the OTA yield that was used as the selection index, the 10^3^ spores/mL group with the most OTA production were used as the representative of low population density, while the 10^6^ spores/mL group with the least OTA production was used as the representative of high population density.

Each strain was cultured on five plates as technical replicates, and each experiment was repeated three times as biological replicates.

### 2.2. Oxylipins Extraction and Detection

Oxidized lipid metabolite extraction and mass spectrometry detection were undertaken using an ultra-high performance liquid chromatography-mass spectrometer (UHPLC-MS) from Biomarker Technologies (Beijing, China). Each sample was accurately determined, and at least 100 mg (dry weight after freeze-drying) was homogenized in 1.2 mL of 70% methanol extracting solution with a ball mill. It was then spiked with 20 µL of 1 µmol/L internal standard mixture to each sample and vortexed for 10 min, centrifuged at 5000 rpm for 10 min at 4 °C, and the extraction was repeated and the supernatants were combined. The oxylipins in the supernatants were extracted using Poly-Sery MAX SPE columns (60 mg, 3 cc, ANPEL, Shanghai, China), the loading solution volume was 3 mL, and they were then rinsed with 2 mL of 20% methanol and finally eluted with 1 mL of 2% acetonitrile formate.

The eluent was dried under vacuum and re-dissolved in 100μL of methanol/water (1:1, *v*/*v*) for UHPLC/MS/MS analysis, and was eluted through an Agilent SB-C18 column (100 mm × 2.1 mm, particle size 1.8 µm) (Agilent, Santa Clara, CA, USA). The solvent system was 0.1% formic acid aqueous solution (A), 0.1% formic acid acetonitrile (B). The injection volume was 4 µL and the gradient was 0~9.0 min from 5% to 95% (B) and maintained for 1 min; 10.0~11.0 min reduced to 5% (B) and equilibrated at 5% to 14 min. The flow rate was 0.35 mL/min and the temperature was 40 °C.

Accurate mass measurements of oxylipin compounds were conducted with a triple quadrupole-linear ion trap mass spectrometer (QTRAP) system equipped with an electrospray ionization (ESI) Turbo Ion-Spray interface, operating in the positive and negative ion mode. The ESI source temperature was 500 °C, the ion spray voltage (IS) was 5500 V (positive) and −4500 V (negative), the ion source gas I (GSI), gas II (GSII), and curtain gas (CUR) were set at 55, 60, and 25.0 psi, respectively, and the collision gas (CAD) was high. Instrument tuning and mass calibration were performed with 10 and 100 µmol/L polypropylene glycol solutions in the QQQ and LIT modes, respectively. All metabolites were analyzed using scheduled multiple reaction monitoring (MRM), and a specific set of MRM transitions were monitored for each period according to the metabolites eluted within this period. The MRM data acquisition and processing were done by using Analyst Workstation Software (Sciex, v1.6.3). The scan mode time of flight (TOF) mass spectra were acquired in negative ion mode by using the TOF at 10,000 mass resolving power for scans over the range from *m*/*z* 100 to *m*/*z* 1200. MS scans were processed using Mass Hunter™ software (version B.02.01, Agilent, Santa Clara, CA, USA). To enhance accurate mass measurement for the ion, a reference solution was vaporised in continuum in the spray chamber. The resulting data were converted to a mass centroid from which the accurate *m*/*z* value was measured.

The absolute concentration of each oxylipin was calculated by comparison to the standard curves generated through the mass spectrometry measure of series dilutions of all 141 oxylipin standards. Only the oxylipins with a signal to noise ratio greater than 10 (S/N > 10) and the concentration located in the range of the standard curves were considered as absolutely quantified. The accuracy and precision of the current method was validated for the linearity of the standard curve, analyte recovery rate, and relative standard deviation (RSD).

### 2.3. Oxidized Lipidomics Analysis

The raw data collected using MassLynx V4.2 was processed by Progenesis QI software for peak extraction, peak alignment and other data processing operations based on the Progenesis QI software online METLIN database and Biomark’s self-built library for identification. After normalizing the original peak area information with the total peak area, principal component analysis and Spearman's correlation analysis were used to judge the repeatability of the samples within group and in addition to the quantity control samples. The identified compounds were searched for classification and pathway information in the KEGG, HMDB, and the LIPID MAPS databases. According to the grouping information, we calculated and compared the difference multiples, and a *t*-test was used to calculate the difference significance pvalue of each compound. The R language package ropls was used to perform OPLS-DA modeling, and 200 times permutation tests were performed to verify the reliability of the model. At the same time, theoretical fragment identification and mass deviation were within 100 ppm, and the oxylipins with Variable Importance in Projection (VIP) > 0.00, Fold Change (FC) > 1, and *p*-value < 1 were considered as significantly changed. The difference metabolites of KEGG pathway enrichment significance were calculated using a hypergeometric distribution test. All data processing and analysis was performed on the biomarker cloud platform (http://www.biocloud.net (accessed on 11 January 2023)).

### 2.4. Transcriptome Analysis

Total RNAs of high/low representative density samples were extracted with a TransZol Up Plus RNA Kit (TransGen Biotech, Beijing, China). RNA concentration and purity was measured using a NanoDrop 2000 (Thermo Fisher Scientific, Wilmington, DE, USA). RNA integrity was assessed using the RNA Nano 6000 Assay Kit of the Agilent Bioanalyzer 2100 system (Agilent Technologies, Santa Clara, CA, USA). Sequencing libraries were generated using the NEBNext^®^Ultra™ RNA Library Prep Kit for Illumina^®^ (San Diego, CA, USA). The sequences were further processed with a bioinformatic pipeline tool, the BMKCloud (www.biocloud.net (accessed on 11 January 2023)) online platform. Transcriptome assembly was accomplished using Trinity with min_kmer_cov set to 2 by default, and all other parameters were set to default. Gene expression levels were estimated by cDNA fragments per kilobase of transcript per million fragments mapped. The differential expression analysis of the two groups was performed using DESeq2. The resulting *p* values were adjusted using Benjamini and Hochberg’s approach for controlling the false discovery rate. Genes with an adjusted *p*-value < 0.05 found by DESeq2 were assigned as differentially expressed. The cluster analysis was conducted by blasting with gene ontology (GO) [30] and the Kyoto Encyclopedia of Genes and Genomes (KEGG) [31]. A functional enrichment analysis (GO and KEGG pathway) was conducted with KOBAS 3.0 [32] software.

### 2.5. Statistical Analysis

Data were expressed as mean ± standard deviation of three independent experiments. All data analyses were performed using the SPSS statistical package (SPSS Version 20.0). A one-way ANOVA with Duncan’s multiple comparison test were used for the analysis of parametric data. Data with different lowercase letters were considered to be significantly different (*p* < 0.05). All assays were replicated in triplicate.

## 3. Results

### 3.1. Qualitative and Quantitative Analysis of Oxylipins with Different Population Density

As a class of lipid secondary metabolites, oxylipins can reflect the diversified information of cell communication and also perform important functions with regard to fungal activities. In order to analyze the effect of population density-mediated environmental changes on *A.ochraceus* from the perspective of lipid metabolism, oxidative lipid metabonomics under representative densities were analyzed. According to the previous results, and considering mycotoxin as the main parameter index [28], the high-density group (10^6^ spores/mL) with low OTA production was selected as the control, while the low-density group (10^3^ spores/mL) was the experimental group, and the qualitative and quantitative analysis of oxylipins was conducted between groups. The oxidized lipid library in this experiment included 141 lipids.

Among PUFAs, LA was the most detected, and reached the level of “10^−1^ μmol/g” in both the high-density and low-density groups, followed by ALA, which reached “10^2^ nmol/g” in each group. ARA, DGLA and DHA reached “10^−1^ nmol/g” in the high-density group, while less in the low-density group. EPA reached “10^−2^ nmol/g” while the low density group was higher than the high-density group. GLA was the least detectable. Overall, with the increase of carbon chain length and unsaturation, the detection amount decreased. Lipid metabolites with a relatively large amount (at least “nmol/g”) are shown in Table 1 (see Table S1 for all detection amount data), and the detected amount of HODEs was the highest among all oxylipins.

It is interesting to note that the shorter the carbon chain length of the PUFA substrate, the greater the production of derived oxylipins. In addition, the amount of oxylipins catalyzed by LOX was greater than CYP and greater than COX. It is speculated that short-chain and low-saturated PUFAs are the substrate of long-chain and high-saturated PUFAs, such as LA is the substrate of ARA, the base amount in cells accounts for the main reason, and the potential energy required for LOX hydroxylation is lower than that of epoxy and double oxygenation.

### 3.2. Density-Mediated Environmental Stress Causes Differences in Lipid Metabolism

**Figure 2:**
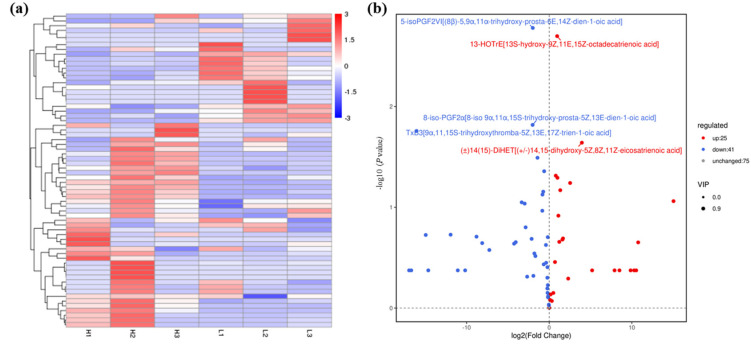
Statistical data of oxidized lipid metabolite levels of *A. ochraceus* with different population densities. (**a**) Oxidized lipid metabolites cluster heat map. (**b**) Volcanic maps of differential metabolites between the high and low density groups. The top five differential metabolites are labeled in the figure, which are 5-isoPGF2VI [(8β) -5,9α,11α-trihydroxy-prosta-6E,14Z-dien-1-oic acid], 13-HOTrE[13S-hydroxy-9Z,11E,15Z-octadecatrienoic acid], 8-iso-PGF2α[8-iso 9α,11α,15S-trihydroxy-prosta-5Z,13E-dien-1-oic acid], TxB3[9α,11,15S-trihydroxythromba-5Z,13E,17Z-trien-1-oic acid], and (±)14(15) -DiHET[(±)14,15-dihydroxy-5Z,8Z,11Z-eicosatrienoic acid]. L represents the low density group, H represents the high density group, and the number indicates three parallels within each group. Red indicates up-regulated metabolite production, and blue indicates down-regulated metabolite production.

To decipher the cell communication mechanism mediated by population density, and if oxylipin acts as QSM to regulate the development in *A. ochraceus*, oxidized lipid metabolomics was performed. Among 141 oxidized lipids, 66 differential metabolites were detected, of which 25 were up-regulated and 41 were down-regulated under the low-density group compared with the high-density control group. The hierarchical clustering of all differential metabolites screened out will cluster with the same or similar attributes in different samples, as has been displayed in the heat maps (Figure 2a). The volcano map shows the distribution of differential metabolites, which can reflect the significant difference between the two groups (Figure 2b). The top five differential metabolites are 5-iso-PGF2VI (ARA, COX), 13-HOTrE (ALA, LOX), 8-iso-PGF_2α_ (ARA, COX), TxB_3_ (EPA, COX), and (±)14(15)-DiHET (EPA, CYP). It can be seen that the difference of long-chain PUFA oxides is more significant, which seems to be related to the complex metabolic processes in the cell, and this also implies a small amount but the efficient utility of complex oxidized lipids in organism activities which are perhaps related to the cascade amplification process.

**Figure 3:**
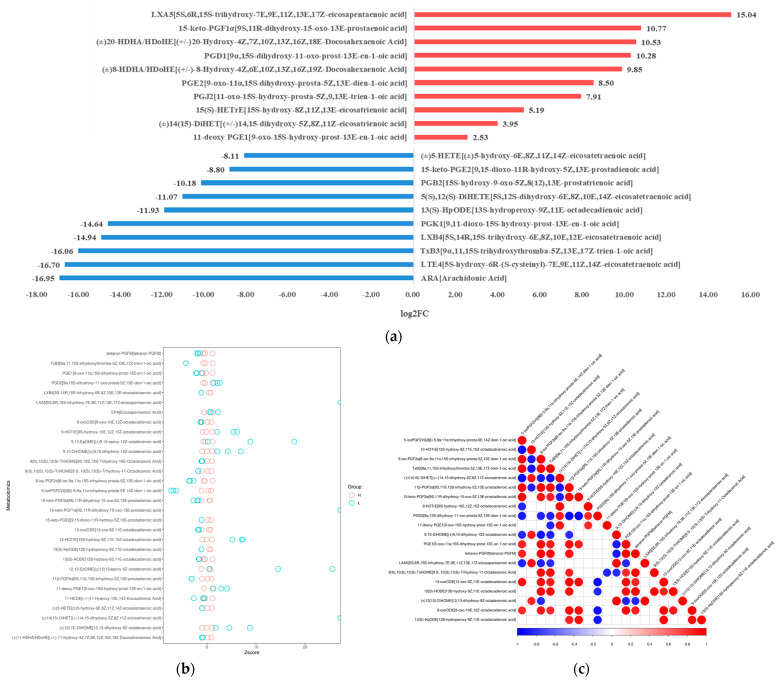
Quantitative analysis of oxidized lipid metabolite levels of *A. ochraceus* with different population densities. (**a**) A statistical chart of the differential metabolite variance. After log conversion of differential metabolite multiples, the log2FC results of the top 10 metabolites up-regulating and down-regulating in the experimental group compared with the control group are shown in the figure. (**b**) The Z-score was calculated based on the mean and standard deviation of the reference data set (control group), and the top 30 differential metabolites sorted by *p*-value are shown in the Z-score plot. (**c**) Differential metabolite correlation analysis. The linear relationship between the two metabolites tends to 1 (red) when they are positively correlated and -1 (blue) when they are negatively correlated.

We compared the fold change of the differential metabolites' quantitative information in the high- and low-density groups (Figure 3a). Significantly up-regulated oxidized lipids mainly included LXA_5_ (EPA, LOX), 15-keto-PGF_1α_ and PGD_1_ (DGLA, COX), HDoHE (DHA, LOX), PGE_2_ and PGJ_2_ (ARA, COX), 15(S)-HETrE (ARA, LOX). Significantly down-regulated metabolic lipids included ARA, LtE_4_ and LxB_4_ (ARA, LOX), TxB_3_ (EPA, COX), 13(S)-HpODE (LA, LOX), and PGB_2_ (ARA, COX). The Z-score is converted from the quantitative value and is used to measure the deviation of the experimental group from the control group, which is another way to visualize the relative content of oxidized lipids (Figure 3b). Metabolites have a synergistic or exclusive relationship. The calculation of the Pearson correlation coefficient between all metabolites and the statistical significance test was carried out for the correlation analysis between different metabolites, and a significance level *p*-value ≤ 0.05 was selected as the threshold for a significant correlation (Figure 3c). There seems to be no specific correlation, because the metabolism and regulatory pathways are too complex; however, in general, most derivatives from the same PUFA are positively correlated. It must be noted that due to the small amount detected, there will be large systematic errors in the data analysis, and further research is needed for a more in-depth analysis.

### 3.3. Analysis of Differentially Expressed Genes Enriched in Various KEGG Pathways

**Figure 4:**
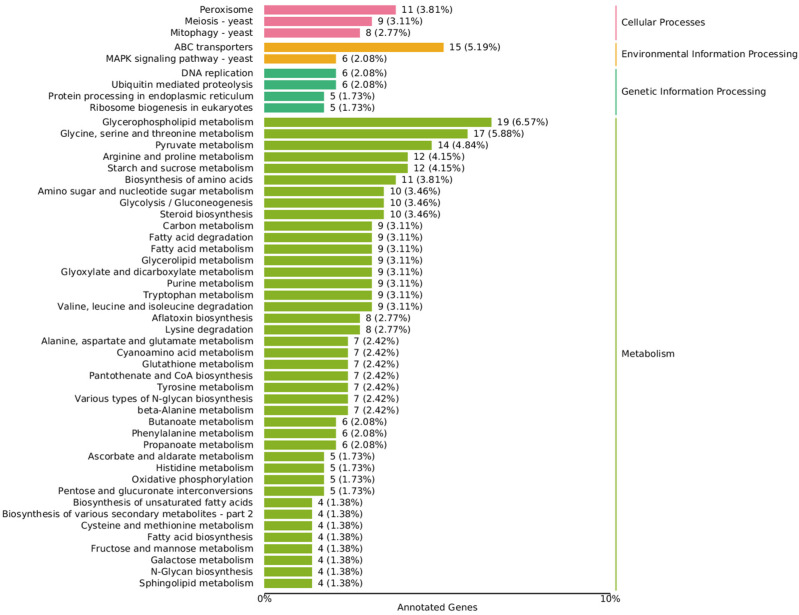
Annotated KEGG classification map of differentially expressed genes. The value is the number of differentially expressed genes, and the proportion of differentially expressed genes in the total number of differentially expressed genes enriched in the pathway is in parentheses.

There is crosstalk and complementarity among various metabolic pathways in fungi, and the results of the metabolome can only reflect the final macroscopic phenomenon, so in order to analyze the causes of density-mediated environmental stress affecting oxidized lipids, a transcriptomic analysis of representative density differentially expressed genes (DEGs) was performed. The annotation results of the DEGs were classified according to the pathway type in KEGG (Figure 4).

In the lipid metabolism class, the fatty acid degradation (ko00071), fatty acid biosynthesis (ko00061), and fatty acid elongation (ko00062) pathways all showed differences between the high and low density groups. The glycerophospholipid metabolism (ko00564) pathway enriched the most DEGs, followed by steroid biosynthesis (ko00100) and glycerolipid metabolism (ko00561), which are two other complex lipids composed of FFAs. Glycerophospholipid, as the basic composition of the cell membrane, is involved in the membrane flow that is responsible for maintaining cell homeostasis and signal transmission. At the same time, the ABC transporters (ko02010) pathway of the membrane transport class and the MAPK signaling pathway (ko04011) of the signal transduction class in the KEGG Environmental Information Processing term had significant differences, which indicate that it is most affected by density-mediated cell communication. Glycerophospholipid releasing FFAs act as raw materials for oxylipins through phospholipase action [17], but the expression of the two phospholipases (TRINITY_DN6235_c0_g1,TRINITY_DN2387_c0_g1), which appeared as the initiation enzymes of PUFA metabolism, did not differ significantly between the high and low density groups.

There were four differentially expressed genes in the biosynthesis of the unsaturated fatty acids pathway (ko01040), accounting for 1.38%, but except for the alpha-linolenic acid metabolism pathway (ko00592) enrichment to two DEGs, most genes in the arachidonic acid metabolism (ko00590) and the linoleic acid metabolism (ko00591) pathway were not affected by density (All gene expression data of lipids metabolism are summarized in Table S2). This may be due to the presence of compensatory pathways to stabilize the metabolism of PUFA. Alternatively, stress from population density (such as ROS) induces non-enzymatic oxidation, resulting in the differential metabolism of PUFAs.

In addition, density epigenetically affected the reproduction and mycotoxin metabolism of *A. ochraceus* [28,33], and it was indeed reflected in gene expression, with the DEGs of the aflatoxin biosynthesis (ko00254) pathway accounting for 2.77%, and the meiosis-yeast (ko04113) pathway accounting for 3.11%. However, compared with the high-density control group, there were both up-regulated and down-regulated genes in the low-density group, and further research on the mechanism of the influence of crosstalk between pathways on the final phenotype needs to be studied further.

### 3.4. Function Annotation of Differentially Oxidized Lipid Metabolites

Complex metabolic reactions and their regulation in organisms are not carried out alone, but often by different genes and proteins to form complex pathways and networks. Differential metabolites interact in organisms to form different pathways, and their mutual influence and regulation eventually leads to systemic changes in the metabolome.

**Figure 5:**
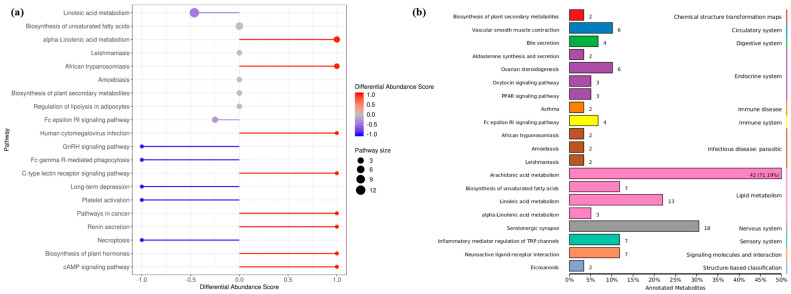
Annotated classification statistical map of differentially oxidized lipid metabolites. (**a**) Differential metabolite abundance score map (**b**) KEGG functional enrichment of all differential metabolites. It consists of twelve primary terms (listed on the right axis) as well as twenty secondary terms (listed on the left axis).

The differential abundance score of oxylipins shows significant changes (including both up-regulation and down-regulation) in various pathways (Figure 5a). Unsaturated fatty acids were greatly affected by the density, LA metabolites were down-regulated, while ALA metabolites were significantly upregulated. In addition, oxidized lipid metabolites involving the biosynthesis of plant hormones and cAMP signaling pathways were significantly up-regulated. Indeed, studies have confirmed the presence of oxylipins in plants with structures similar to those in fungi, and interspecific interactions are regulated by activating the production of cAMP. The KEGG database was used to annotate the differential metabolites, and the TOP20 entries with the most differential enrichment pathways were selected (Figure 5b). As a long-chain and highly unsaturated PUFA, the ARA metabolic pathway contained the most derivatives, and the proportion of differential metabolites was as high as 71.19%. In addition, differential metabolites were involved in the endocrine and immune system, which seems to hint at the role of oxylipins in fungal host infection.

### 3.5. Inference of Oxylipins as Density-Mediated QSM in Aspergillus ochraceus

**Figure 6:**
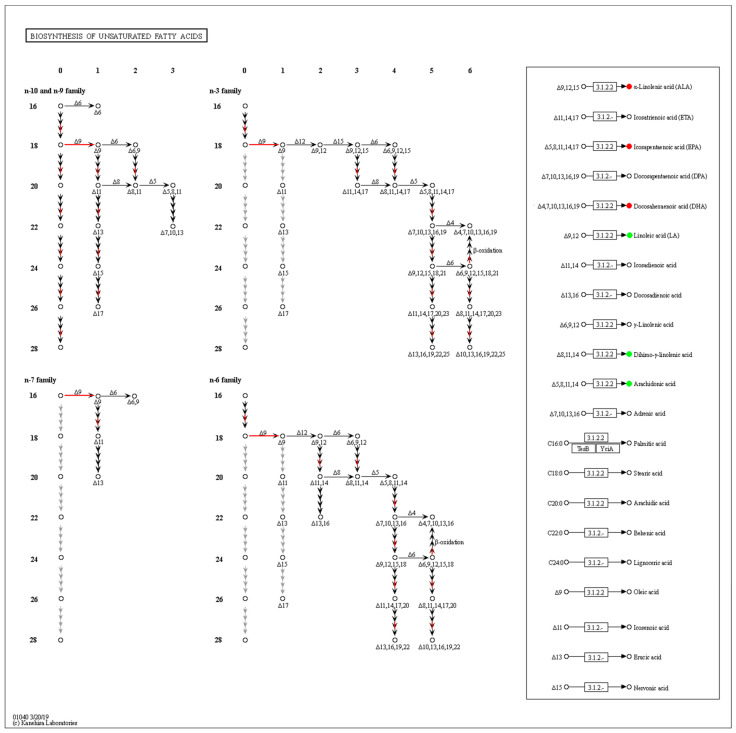
Enrichment of differentially expressed genes and differentially oxidized lipid metabolites in the biosynthesis of the unsaturated fatty acids pathway. DEGs are shown on the left side, the abscissa is the number of double bonds, the ordinate indicates the number of carbon atoms in the skeleton, and the red arrow indicates the upregulation of gene expression. The box on the right shows the differential metabolism of the main PUFAs, with red circles indicating upward metabolites and green circles indicating downward metabolites.

Combined with the transcriptomic analysis, the biosynthesis of the unsaturated fatty acids (ko01040) pathway was greatly affected by the fungi density, and the expression of related genes was significantly up-regulated in the low-density group. The amounts of ALA, EPA and DHA increased, while LA, DGLA and ARA decreased significantly (Figure 6). There are further metabolic processes, so we cannot conclude that these PUFAs play decisive roles in QS, and thus the metabolic analysis of each PUFA was conducted.

**Figure 7:**
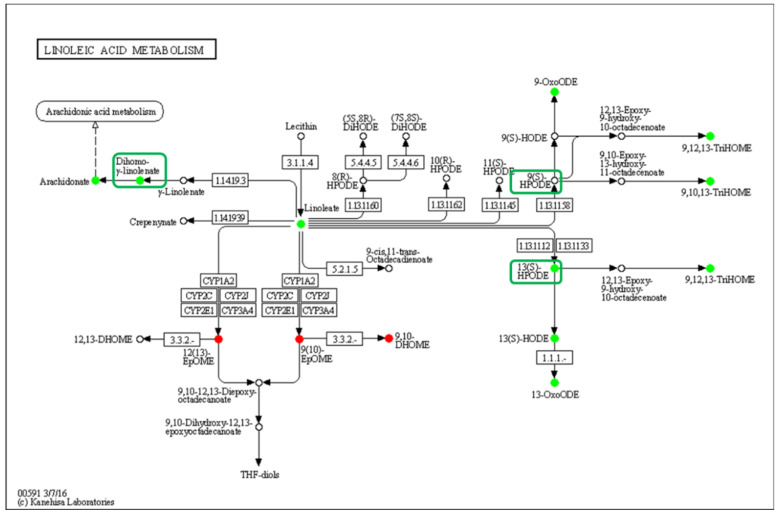
Enrichment of differentially oxidized lipid metabolites in the linoleic acid metabolism pathway (ko00591). No differentially expressed genes were detected, and differential metabolites are circled, while boxes highlight the elucidated signaling molecule HODEs.

The linoleic acid metabolism pathway was significantly down-regulated, and 13(S)-HPODE and 13(S)-HODE, which have been verified as QSM, and their derivatives were significantly reduced in the low-density group, while the other QSM, 9-HODE, had no significant change, but its derivatives were significantly reduced (Figure 7). Curiously, the DEGs in this pathway showed that the gene expression was not affected by density, which indicates that LA oxidation was affected by stress caused by population density rather than by the action of intracellular oxidases.

**Figure 8:**
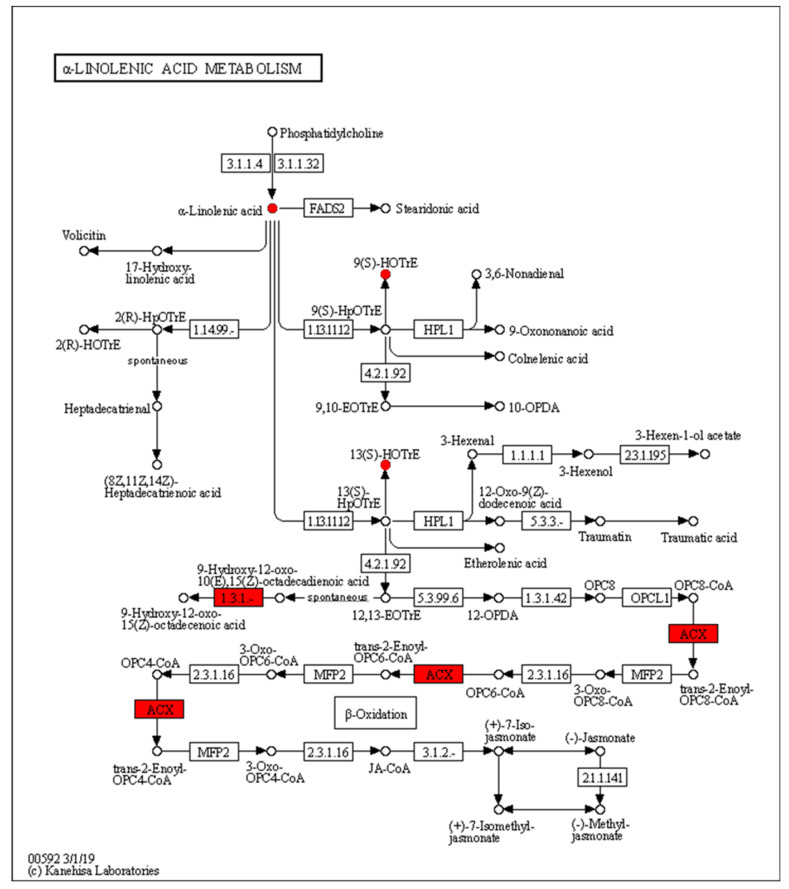
Enrichment of differentially expressed genes and oxidized lipid metabolites in the alpha-linolenic acid metabolism pathway (ko00592). Upregulated differentially expressed genes are marked with red boxes, and upregulated differentially expressed metabolites are marked with red circles.

**Figure 9:**
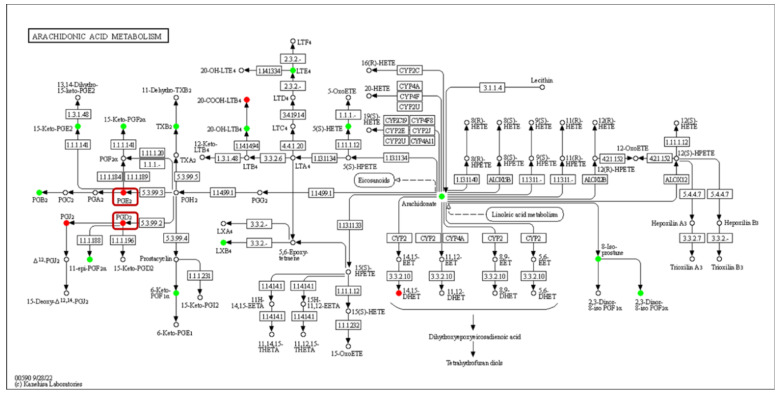
Enrichment of differentially oxidized lipid metabolites in the arachidonic acid metabolism pathway (ko00590). No differentially expressed genes were detected, and differential metabolites are circled, while boxes highlight the novel signaling molecule PGs.

The expression of acyl-CoA oxidase gene (ACX, EC:1.3.3.6) in the alpha-linolenic acid metabolism was upregulated, but seems to be unassociated with the increased production of 9(S)-HpOTrE and 13(S)-HpOTrE (Figure 8). The ARA metabolic pathway was enriched with the most differential metabolites, and seems to be most affected by density. Among ARA metabolites, the detected amounts of most metabolites were down-regulated at low density, except that PGE_2_ and PGD_2_,which are substrates of the prostaglandin D2 receptor (PTGDR) and prostaglandin E2 receptor (PTGER) in the neuroactive ligand-receptor interaction (ko04080) pathway (Figure 9), increased. However, it must be noted that due to the small intrinsic content of ARA and its derivatives, a tiny quantitative change will cause a statistically significant difference. Therefore, even if the macro showed that ARA metabolism is closely related to density, it must be confirmed further. Fortunately, this also expanded the range of oxylipins as QSM in *Aspergillus*.

This shows that under the influence of population density, firstly, the metabolism of various PUFAs are affected, and the synthesis mechanisms of oxylipins are difficult to speculate upon. Furthermore, whether single specific or multiple substances play the role of QSM needs further research. Third, it is worth exploring at which level and what role other pathways are affected by density in *A. ochraceus*.

## 4. Discussion

Fungi have strong adaptability and exhibit rapid aggregation growth under appropriate conditions, while producing secondary metabolites that are beneficial or harmful, and how microorganisms survive the environmental pressure caused by population density is a comprehensive mechanism. The fatty acids' oxidative products not only regulate fungi intraspecific communication, but it was further found that the production and function of oxylipins are interoperable in the interaction between fungi and hosts. That is, oxylipins are a class of widespread signaling molecules among animals, plants, and microorganisms [34]. Although there are only a few unsaturated fatty acids as substrate and specific oxidase families, a large family of oxylipins has been derived due to the variety of processing sites and catalytic forms. In *Aspergillus*, the PUFAs and their catalytic products can regulate the morphological transformation and secondary metabolism to affect their environmental adaptability and infectivity to the host [35]. Based on the specific structural recognition of signal molecules as ligands by receptors, it is necessary to clarify their synthesis mechanism and further analyze their function to study the regulation of pressure signals transmitted by QSMs on the development of *A. ochraceus*.

Fatty acids play an extremely important role in cellular life activities and also serve as substrates for complex biomacromolecules, such as existing in the cell membrane in the esterified form, maintaining cell structure, and coordinating the transport of intracellular and extracellular substances, or being further processed into various derivatives. The metabolism of FAs starts from de novo synthesis with acetyl-CoA as the raw material, while also including elongation and degradation, which is comprehensively regulated by various factors [17]. In addition, the oxidase and substrate do not strictly correspond on a one to one basis, as there is crosstalk and switching between oxidation pathways [20]. Therefore, the detection amount of the same oxylipin under diverse conditions is different, and the content of different oxylipins varies greatly, which results in poor repeatability or even contradictions when compared with previous research results. However, considering that the efficiency threshold of oxylipins differ greatly, for example, the concentration of PGs that can modulate *A. fumigatus* development is only “nmol” level, while HODEs reached the “mmol” level [36], so the exploration of oxylipins should not be limited to specific types.

Psi catalyzed by PPOs are divided into PsiA, PsiB, and PsiC according to the positioning of the hydroxy groups. PsiAα enhances asexual reproduction, while PsiBα and PsiCα stimulate sexual reproduction, and the ratio of PsiAα to PsiBα and PsiCα determines the form of fungi reproduction [37,38,39]. The overexpression of *ppoA* in *A. nidulans* reduced the ratio of asexual to sexual reproduction, the *ΔppoC* of *A. fumigatus* decreased the spore germination rate, but the size of the spore enlarged, double the *∆ppoAC* and triple the *∆ppoABC* mutants of *A.nidulans* delayed and reduced conidiospores development and turned to a premature increase in ascospore production. LOXs catalyze the oxidation of LA to produce HpODEs, which are quickly hydroxylated to HODEs, both of which can affect vegetative growth, morphological transformation, and mycotoxin synthesis through GPCRs, which then activate the cAMP-PKA pathway and regulate the expression of genes. For example, In *A. nidulans*, *A. fumigatus* and *A. flavus*, 5,8-diHODE and 7,8-diHODE induce cell differentiation and lateral branching, and also regulate sexual development and sporulation [19,35,40]. The knockout of all dioxygenase genes (*ppoA*, *ppoB*, *ppoC* and *ppoD*) and one lipoxygenase gene (*loxA*) in *A.flavus* destroyed its density-sensing ability, produced high levels of aflatoxin (AF) at any density, as well as during infestation with corn and peanut, and shifting from asexual to sexual development [25]. In *A. ochraceus*, the Δ*lox* mutant that the 13-HODE synthesis inhibited, OTA production decreased and conidia production increased [41]. The addition of exogenous 9-HODE to wild type *A. ochraceus* inhibited the sporogenesis and promoted OTA synthesis, which indicated the state of low population density, while the effect of 13-HODE was the opposite, and this revealed the effect of HODEs as QSM [28].

However, the quantitative analysis results in this experiment showed that, compared with the high-density group, the detection of 9-HODE and 13-HODE in the low-density group were both reduced, and the previous experiments also showed that a relatively larger ratio of 9-HODE was produced in a low population density [33]. It is hypothesized that the smaller amount of HODEs detected in low density was mainly due to fewer individual cells, although a single cell may produce more HODEs. In addition, we venture a guess that these two HODEs may competitively bind to membrane receptors, and GPCR may be more sensitive to 9-HODE even if the total amount of HODEs in the environment is reduced. In *A. nidulans*, the addition of 9- and 13-HODE in wild-type induced cAMP surged, but the *ΔgprC* and *ΔgprD* mutants remained unchanged [42]. A library of a total of 15 *gpr* gene mutations were constructed in *A. flavus*, while with the addition of LA, the raw material of HODEs, only the *ΔgprD* mutant showed phenotypic differences from the wild type, but the added13-HODE had nine GPCRs deletion mutant strains, including *ΔgprD*, that did not show the same increase in spore production as WT [43]. Additional studies of oxylipin function have been comprehensively summarized in the review [44,45]. These results indicate that there are a variety of GPCRs in *Aspergillus* with oxylipins as ligands, and the identification of receptors requires specific structures. A bioinformatics analysis of GPCRs was also carried out in *A. ochraceus*, and a sequence comparison identified GPCR receptors that may be bound by oxylipins [46], which can be verified through gene knockout and complement. Positively, transcriptomics showed that the cAMP pathway was significantly up-regulated at low density, and, unexpectedly, the MAPK pathway was also significantly different, indicating that the adaptability of *A. ochraceus* to environmental signals was comprehensively regulated by multiple intracellular effectors [24].

In the process of fungal infection, both the fungus and the hosts are under stress and coercion brought by each other, and their respective defense systems are activated through oxylipins [47,48]. Oxidases that belong to the same family of animals, higher plants, and fungi have homologous domains; for example, the wild-type phenotype can be restored through the insertion of maize lipoxygenase in *Aspergillus ΔppoA* and *ΔppoC* mutants [49]. In addition, oxylipins play a reciprocal ecological role with each other. Fungal oxidases can catalyze raw materials of the host for synthesizing oxylipins that act as developmental regulators and enhance their own infection capacity, including the production of more conidia and mycotoxin or switching to the biofilm stage [50]. Conversely, plants can use fungal oxylipins as precursors of their own oxylipins that act as defense regulators to activate resistance to fungal infections [51,52]. Maizes with the *lox* knockout that lost the ability to produce oxylipins, such as JA and its derivatives, were more susceptible to *A. flavus* and *A. nidulans*, which produced more spores and mycotoxins [53,54]. Human pathogen *A. fumigatus* and *A. nidulans* can transform ARA into PGE_2_, PGF_2α_, 6-keto-PGF_1α_, isoprostanes, and TxB_2_, which is similar to mammal hosts, but the pathways for the synthesis of these compounds are different [36,55]. There are no more relevant studies of PGs in *Aspergillus*, perhaps because PGs are tiny, have a short half-life, and are unstable, and the measured amount in this experiment is only 10^−2^ times that of the HODEs. However, it should be noted that the detected amount of PGs was significantly up-regulated at low density, considering that PGs is an important class of GPCR ligand in the mammal, and the scope of QSM in *Aspergillus* can be expanded to PGs and other ARA derivatives in the future [44]. In conclusion, although there is no commercial application for oxylipins, the interruption of the fungal communication system from the perspective of quorum sensing signal transmission may be a potential means of biological control of fungal pollution in the future.

## 5. Conclusions

This paper focused on the lipid metabolomics combined with transcriptomic profiles in *A. ochraceus* under low- and high-densities, and explored the underlying QS mechanism of *Aspergillus* to population stresses. Under low-density, *A. ochraceus* produced fewer conidia and more mycotoxin OTA, while under high density, which involved resource stress and high pressure, OTA synthesis decreased and there was sexual switching to asexual reproduction. Population density mainly affects the lipid metabolism and environmental information processing in *A. ochraceus*. The G protein signaling pathway and the cAMP pathway showed significant differences, which are related to the regulation of fungal morphology and the secondary metabolism. Differential lipid metabolites, including the elucidated HODEs and novel potential PGs, may perform the function of QSM. However, in addition to being catalyzed by LOX, COX, CYP, and other enzymes, the synthesis of oxylipins also appears to be non-enzymatic due to oxidative stress caused by high population density. The series of oxylipins produced are recognized by different receptors based on structural differences, and regulate the fungal development and the interaction with the plant and animal hosts in the form of signaling molecules, constituting a complete QS communication mechanism. Subsequently, the effect of HODEs or PGs on fungal sporulation and mycotoxin biosynthesis can be verified by additional experiments, which is likely to lead to new biological methods of fungal damage control. Even further verification of key links such as oxidases, GPCRs, and cAMP effectors is required at the gene level in order to complete the mysterious and complex intraspecific and interspecific communication mechanisms.

## Figures and Tables

**Table 1:** The amount of unsaturated fatty acids and oxidized derivatives detected in the low- and high-density groups.

Lipid Type	Full Name	Abbreviation	Substrate	Enzyme	High-Density (nmol/g)	Low-Density (nmol/g)
PUFAs	Linoleic Acid	LA	-	-	293.39	222.72
	α-Linolenic Acid	ALA	-	-	28.10	30.12
	γ-Linolenic Acid	GLA	-	-	/	/
	Dihomo-γ-Linolenic Acid	DGLA	-	-	0.50	0.13
	Arachidonic Acid	ARA	-	-	0.38	/
	Docosahexaenoic Acid	DHA	-	-	0.12	0.15
	Eicosapentaenoic Acid	EPA	-	-	0.01	0.04
Oxylipins	(±)-9-hydroxy-10E,12Z-octadecadienoic acid	(±)9-HODE	LA	LOX	3.79	3.38
	13S-hydroxy-9Z,11E-octadecadienoic acid	13(S)-HODE	LA	LOX	2.76	1.54
	9S,12S,13S-Trihydroxy-10E-Octadecenoic Acid	9(S),12(S),13(S)-TriHOM	LA	LOX	6.12	1.40
	(±)9,10-epoxy-12Z-octadecenoic acid	9,10-EpOME	LA	CYP	0.23	0.54
	(±)12(13)epoxy-9Z-octadecenoic acid	12,13-EpOME	LA	CYP	0.15	0.48
	9(S),10(S),13(S)-Trihydroxy-11-Octadecenoic Acid	9(S),10(S),13(S)-TriHOME	LA	LOX	1.19	0.12
	12,13-dihydroxy-9Z-octadecenoic acid	(±)12(13)-DiHOME	LA	CYP	0.05	0.10
	(±)9,10-dihydroxy-12Z-octadecenoic acid	9,10-DiHOME	LA	CYP	0.03	0.08
	13S-hydroxy-9Z,11E,15Z-octadecatrienoic acid	13-HOTrE	ALA	LOX	0.26	0.50
	8-iso 9α,11α,15S-trihydroxy-prosta-5Z,13E-dien-1-oic acid	8-iso-PGF_2α_	ARA	COX	0.97	0.24
	9α,11α,15S,19R-tetrahydroxy-prosta-5Z,13E-dien-1-oic acid	19(R)-hydroxy PGF_2α_	ARA	COX	0.15	0.13
	9α,11,15S-trihydroxythromba-5Z,13E-dien-1-oic acid	TxB_2_	ARA	COX	0.10	0.09
	5S,12R-dihydroxy-6Z,8E,10E,14Z-eicosatetraene-1,20-dioic acid	20-COOH-LtB_4_	ARA	LOX	0.03	0.05
	9α,15S-dihydroxy-11-oxo-prosta-5Z,13E-dien-1-oic acid	PGD_2_	ARA	COX	0.02	0.05
	5S,6R,15S-trihydroxy-7E,9E,11Z,13E,17Z-eicosapentaenoic acid	LxA_5_	EPA	LOX	/	0.10

Notes: The values are the average of three parallel samples within the group, the unit is the mole of oxylipin detected per gram of mycelium; “/” indicates that the amount value was not detected; “-” indicates that this content was not involved for the time being.

## Data Availability

Not applicable.

## Supplementary Materials

The following supporting information can be downloaded at: https://www.mdpi.com/article/10.3390/metabo13040491/s1, Table S1: The detection amount of each lipid; Table S2: The expression levels of oxygenases genes.

## Abbreviations

ALA: α-linolenic acid; ARA, arachidonic acid; COX, cyclooxygenase; CYP, cytochrome P450 enzyme; DEG, differentially expressed gene; DGLA, dihomo-γ-linolenic acid; DHA, docosahexaenoic acid; DOX, dioxygenase; EPA, eicosapentaenoic acid; FDA, food and drug administration; FFA, free fatty acid; GRAS, generally recognized as safe; HODE, hydroxyoctadecadienoic acid; IARC, International Agency for Research on Cancer; LA, linoleic acid; LDS, linoleate dioxygenase; LOX, lipoxygenase; MUFA, monounsaturated fatty acid; OA, oleic acid; OTA, ochratoxin A; PDA, potato dextrose agar; PG, prostaglandin; PPO, psi producing oxygenase; psi, precocious sexual inducer; PTGDR, prostaglandin D2 receptor; PTGER, prostaglandin E2 receptor; PUFA, polyunsaturated fatty acid; QS, quorum sensing; QSM, quorum sensing molecule; ROS, reactive oxygen species; UHPLC-MS, ultra-high performance liquid chromatography-mass spectrometry.
